# Otitis media with effusion: Accuracy of tympanometry in detecting fluid in the middle ears of children at myringotomies

**DOI:** 10.12669/pjms.322.9009

**Published:** 2016

**Authors:** Khurshid Anwar, Saeed Khan, Habib ur Rehman, Mohammad Javaid, Isteraj Shahabi

**Affiliations:** 1 Khurshid Anwar, Senior Registrar, Department of ENT and Head & Neck Surgery, PGMI Hayatabad Medical Complex, Peshawar, Pakistan; 2 Saeed Khan, Trainee Medical Officer, Department of ENT and Head & Neck Surgery, PGMI Hayatabad Medical Complex, Peshawar, Pakistan; 3 Habib ur Rehman, Assistant Professor, Department of ENT and Head & Neck Surgery, PGMI Hayatabad Medical Complex, Peshawar, Pakistan; 4 Mohammad Javaid, Associate Professor, Department of ENT and Head & Neck Surgery, PGMI Hayatabad Medical Complex, Peshawar, Pakistan; 5 Isteraj Shahabi, Professor & Head, Department of ENT and Head & Neck Surgery, PGMI Hayatabad Medical Complex, Peshawar, Pakistan

**Keywords:** Accuracy, Hearing impairment, Otitis Media with Effusion, Tympanometry

## Abstract

**Objective:**

(1) The diagnostic accuracy of tympanometry in detecting fluid in the middle ear space in children with otitis media with effusion by comparing its findings with those of myringotomies. (2) Identify the age group most commonly affected by OME.

**Methods:**

This prospective study was conducted at the Department of ENT& Head and Neck Surgery, Postgraduate Medical Institute Hayatabad Medical complex, Peshawar from July 1, 2012 to April 30, 2015. Patients with suspicion of OME underwent tympanometry and later myringotomies. Using Jerger’s classification, Type B tympanogram with normal canal volume was considered as conclusive evidence of fluid in the middle ear space. Its findings were compared with those of the respective myringotomies. From the data collected, the accuracy, sensitivity, specificity, positive predictive value and negative predictive values were calculated.

**Results:**

A total 117 ears of 63 patients were operated. The age range was 3 to 12 years. The commonest age group (58.7%) affected by OME was 6-8 years. Type B tympanogram with flat curve and normal canal volume was obtained in 71.4% of the ears. Comparison with myringotomy findings showed TP 85, TN 13, FP 5 and FN 14. The diagnostic value of tympanometry was; Sensitivity 85.85%, Specificity 72.22%, PPV 94.44%, NPV 48.14% and Accuracy of 83.76%. P value calculated using chi square test showed that there was significant difference between tympanometry and myringotomy findings in OME (p < 0.05).

**Conclusions:**

OME is common in age group 6-8 years. Tympanogram Type B with normal canal volume is fairly sensitive in diagnosing this condition. However for occurrence of false positive results, final decision regarding management should be made on clinical findings and other supportive audiological tests.

## INTRODUCTION

Otitis media with effusion (OME) is defined as fluid in the middle ear and, sometimes, the mastoid air cell system without signs or symptoms of ear infection.^1^ Symptoms usually involve hearing loss or aural fullness but typically do not involve pain or fever. The condition is said to be chronic when the fluid accumulation persists beyond 12 weeks.^2^

OME occurs commonly during childhood, with as many as 90 percent of children (80% of individual ears) having at least one episode of OME by age 10.^3^ Unlike acute otitis media, the prevalence of chronic otitis media with effusion is unknown. Several studies have reported various estimates of the condition according to age. Rates vary from 13% at age one year, 14% at age two years, 10% at age three years and 2.8% among children aged 7-8 years.^4^-^6^ The resultant hearing impairment has its own sequelae. However its long-term impact on child developmental outcomes such as speech, language, intelligence, and hearing remains unclear. Children with Eustachian tube dysfunction, adenoid hyperplasia, nasal allergy, cleft palate, Down syndrome and other craniofacial anomalies are at high risk for developing OME.^7^ Recently GERD has also been implicated in OME in young children.^8^ Although rare, OME also occurs in adults. This usually occurs following upper respiratory infection, severe nasal allergies and rapid air pressure changes during flight or scuba diving. The incidence of prolonged OME in adults can occur but is rare and is much less common than in children.^9^

Correct diagnosis is vital for the management of children with OME. The clinical diagnosis of OME is made by history, otoscopy, pneumatic otoscopy and impedance audiometry. The otoscopic findings in OME are mainly different combinations of retraction of the pars tensa and wide variations colour of the tympanic membrane. Tympanometry provides useful quantitative information about the presence of fluid in the middle ear, mobility of the middle ear system, and ear canal volume. Its use has been recommended in conjunction with more qualitative information (e.g.history, appearance and mobility of the tympanic membrane) in the evaluation of otitis media with effusion.

A type B tympanogram with flat curve and normal canal volume is considered diagnostic of OME. Compared with all other types of tympanograms it has a sensitivity of between 56 and 73 percent and a specificity of between 50 and 98 percent in detecting OME confirmed surgically.^10^ The aim of our study was to assess the accuracy of this particular type of tympanogram in OME.

## METHODS

This prospective study was conducted at the Department of ENT& Head and Neck Surgery, Postgraduate Medical Institute Hayatabad Medical complex, Peshawar from July 1, 2012 to April 30, 2015. It was a prospective and comparative study using the non-probability convenience sampling technique.

### Sample Size

The study included 63 cases with tympanometric evidence of OME undergoing myringotomies during this period. Sample size was calculated using 13% proportion of Otitis media with effusion, 95% confidence level and 5% margin of error using WHO software for sample size determination.

### Inclusion Criteria

1. Patients between the age of 3-12 years and belonging to both the sexes.
2. All patients undergoing tympanometry followed by myringotomies.
3. Patients with types A, B and C tympanograms with clinical evidence of OME.
4. Pure tone audiometry showing conductive hearing loss with A-B gap of >30 dB in better ear at first visit.

### Exclusion Criteria

1. Ears with otoscopic evidence of tympanosclerosis.
2. Patients with frankly discharging ears or having evidence of cholesteatoma.
3. Patients with Type B tympanograms with flat curve and above normal canal volume.
4. OME persisting for less than 3 months.

### Data Collection Procedure

Patients fulfilling the laid down criteria were included in the study. The procedure was explained and Informed consent obtained from the parents. Ethical approval for the study was obtained from the institutional ethical committee. A detailed history was obtained regarding hearing impairment, its duration & mode of onset, progression of symptoms and performance at school. Further enquiry was made to look for the presence or otherwise of nasal allergies, recurrent episodes of upper respiratory tract infections, cleft palate repair, snoring & sleep disturbances and concomitant systemic disorders. A detailed ENT and systemic examination including indirect nasopharyngoscopy were undertaken. Otoscopic examination was focused to look for signs of OME. Abnormal coloration and retraction of tympanic membrane, air bubbles or fluid level in the middle ear cavity were taken as positive signs of OME. Whisper test and conversational voice test, where appropriate, were used to estimate roughly the hearing impairment. Tuning fork tests such as Rinne, Weber and Absolute Bone Conduction tests carried out to determine the type of hearing loss. Patients with clinical suspicion of OME underwent tympanometry. The tympanograms were obtained from different centers but all using 226 Hz probe tone. Normal ear canal volume was taken as 0.3 - 1 mL and the curve was considered flat when it had no discernible peak over a pressure range of +200 daPa to -400 daPa. Pure Tone Audiogram (PTA) was also obtained in selected children. X-ray films of the nasopharynx for adenoids were obtained in cases suspected of having adenoid hypertrophy. The tympanograms were classified using Jerger’s classification as: 1. Type A 2. Type B (flat curve and normal canal volume) and 3. Type C. Patients with suspected OME were booked for myringotomies and any other concomitant surgery such as adenoidectomy. Haematological and other relevant investigations were carried out to determine patients’ suitability for surgery and fitness for general anaesthesia. All myringotomies were carried out through a radial incision in the antero-inferior quadrant using a general inhalational anaesthetic agent. Type B tympanogram with flat curve and normal canal volume alone was taken as conclusive evidence for the presence of fluid in the middle ear space. The operative findings at myringotomy were recorded and thus labeled as: I. True Positive (TP) when fluid was present and II. False Positive (FP) when no fluid was aspirated. In cases where the tympanograms were either Type A or Type C, the findings were categorized as: III. True Negative (TN) when no fluid was aspirated and IV. False Negative (FN) when fluid was present. The accuracy, sensitivity, specificity, positive predictive value (PPV) and negative predictive value (NPV) of Type B tympanogram with flat curve and normal canal volume were calculated using the following formulae:Accuracy = (TP+TN) ×100/(TP+TN+FP+FN), Sensitivity = (TPx100) / (TP+FN), Specificity = (TNx100) / (TN+ FP), PPV = (TPx100) / (TP+ FP) and NPV= (TNx100) / (TN+ FN).

### Statistical Analysis

The data was recorded on a proforma and the descriptive statistics were analyzed using SPSS 16 for Windows to determine frequencies for variables like gender, age, duration of symptoms, types of tympanograms and myringotomy findings. Chi- square test was applied to determine the significance of findings at tympanometry and myringotomies.

## RESULTS

The study included 43 males and 20 females and a total 117 ears of 63 patients were operated. The M: F ratio was 2.15:1. The age range was 3 to 12 years with mean age of seven years and a standard deviation of +/-2.124. The commonest age group (58.7%) affected by OME was 6-8 years. The majority (47.6%) of children undergoing myringotomy had OME persistent for 6-12 months followed by those (25.4%) who had it for more than one year (Table-I). Type B tympanogram with flat curve and normal canal volume was obtained in 71.4% of the ears examined. The frequency and types of tympanograms obtained in the Left and Right ears are shown in Table-II. On the right side there were 68.3% TP, 12.7% TN and 11.1% FN as shown in Table-III. On the left side there were 66.7% TP, 8% TN, 8% FP and 11% FN as depicted in Table-IV. Out of the total 9 non- operated ears, 4 occurred on the left side. The tympanometry and myringotomy findings crosstables for both the left and right ears and Chi-square test applied showed that with respect to determination of fluid in the middle ear, there was significant difference between tympanometry and myringotomy findings on both the left and right sides (p <0.05).. The diagnostic value of tympanometry calculated was; Sensitivity 85.85%, Specificity 72.22%, PPV 94.44%, NPV 48.14% and overall Accuracy of 83.76%.

**Table-I:** Age groups and duration of symptoms

		Frequency	Percent	Cumulative Percent
Age	3-5	13	20.6	20.6
	6-8	37	58.7	79.4
	9-12	13	20.6	100.0
	Total	63	100.0	
Duration of symptoms	3-6 months	17	27.0	27.0
	6-12 months	30	47.6	74.6
	> 1 year	16	25.4	100.0

	Total	63	100.0	

**Table-II:** Tympanogram Types.

		Frequency	Percent	Cumulative Percent
Left Tympanogram Types	Type A	3	4.8	4.8
	Type B	47	74.6	79.4
	Type C	13	20.6	100.0
	Total	63	100.0	
Right Tympanogram Types	Type A	8	12.7	12.7
	Type B	43	68.3	81.0
	Type C	12	19.0	100.0
	Total	63	100.0	

**Table-III:** Comparison of tympanogram and myringotomy findings (Right Ear)

			Rt Myringotomy findings			Total

			Ear not operated	Fluid present	Fluid absent	
Rt Tympanogram Type	Type A	Count	2	0	6	8
		% of Total	3.2%	0.0%	9.5%	12.7%
	Type B	Count	0	43	0	43
		% of Total	0.0%	68.3%	0.0%	68.3%
	Type C	Count	3	7	2	12
		% of Total	4.8%	11.1%	3.2%	19.0%
Total		Count	5	50	8	63
		% of Total	7.9%	79.4%	12.7%	100.0%

Calculated p-value for the Right Ear=0.000

**Table-IV:** Comparison of tympanogram and myringotomy findings (Left Ear)

			Lt Myringotomy findings			Total

			Ear not operated	Fluid present	Fluid absent	
Lt Tympanogram Type	Type A	Count	1	0	2	3
		% of Total	1.6%	.0%	3.2%	4.8%
	Type B	Count	0	42	5	47
		% of Total	.0%	66.7%	7.9%	74.6%
	Type C	Count	3	7	3	13
		% of Total	4.8%	11.1%	4.8%	20.6%
Total		Count	4	49	10	63
		% of Total	6.3%	77.8%	15.9%	100.0%

Calculated p-value for the Left Ear=0.000

## DISCUSSION

Otitis media with effusion (OME) is a common but treatable cause of deafness in children. It leads to delay in speech acquisition, behavioral problems and poor performance at school depending on the age at which it affects the child. There is a need to diagnose it correctly at an earlier stage. Tympanometry in conjunction with history and clinical examination is the method most commonly used. It has been confirmed as sensitive and fairly specific in identifying children with material hearing loss associated with OME. The affected children should be observed closely by serial tympanometry as some 50% of such cases resolve after three months and do not justify further management unless the condition recurs. Exceptions to this policy are those children having a pure-tone average in the better ear of > 30 dB HL at their first visit. In these children the probability of persistence of OME is greater than 80%.^11^

The data on prevalence of OME is highly varied in the literature and in our country the literature is scarce on the subject. A study conducted at the Holy Family Hospital by Tallat Najeeb and colleagues using otoscopy and tympanometry found OME in 7% of the 563 children examined.^12^ Another study was conducted by Tallat Jabeen and colleagues in the twin cities of Rawalpindi and Islamabad involving 600 children in different schools. Using tympanometry as a screening tool, they found OME in 13% of these children. Type B curve was found in 88.5% and type C curve was obtained in 11.5% of these children.^13^ These findings are in contrast to those of our study. The types of tympanograms types obtained in our patients were; Type B (flat curve, normal canal volume) 71.4%, Type C 19.84% and Type A in 8.7%. Analyzing papers with the findings at myringotomy as the reference ’gold’ standard, suggest that a type B tympanogram is the most frequently obtained type in OME, a type A is infrequently associated with OME and a type C falls in between.^14^ These findings are in agreement with our current study.

Age and climate are well known factors that influence the occurrence of OME. OME is usually found in the relatively younger 3-5 years age group. In a large study in China involving 2902 children aged 2-8 years, the point prevalence of OME was 4.3%. By age group, the findings were 14.0% in two years old, 8.3% in 3 years old, 5.0% in 4 years old, 4.9% in five years old, 2.8% in 6 years old, 1.7% in 7 years old, and 3.2% in eight year old.^15^ Our findings that the 6-8 years age group was most commonly affected are supported by Okur E and colleagues who in their study involving 2930 children found the highest point prevalence of 10.4% in the same age group.^16^ In a Nepalese study by Mark A and colleagues found the peak age affected was 10 years (23.1%) in contrast to the findings of this and the Chinese study.^17^

A Turkish study using confirmation of middle ear effusion by myringotomy as the gold standard, found that tympanometry had sensitivity of 96% and a positive predictive value of 92%. There was a false positive rate of 8 percent.^18^ Five false positive cases occurred in our study. One possible explanation could be the fact that inhalational anaesthetic can itself aerate the middle ear giving a ’false’ dry tap.^10^ A similar study from Mosul, Iraq, using fluid tap at myringotomy as gold standard and the type B tympanogram with flat curve as indicative of OME reported an accuracy of 71.4%, sensitivity 97.3%, specificity 57.2%, positive predictive value 55.3% and negative predictive value 97.5%. The Type B flat curve was obtained in 62% of these patients.^19^

## CONCLUSIONS

OME is more common in age group 6-8 years. Tympanometry with a resultant Type B flat curve with normal canal volume is fairly sensitive and reliable technique in diagnosing this condition in children aged 3-12 years. However due to occurrence of false positive results, final decision regarding management of such children should be made on clinical findings and other supportive audio logical tests.
